# Serum Early Prostate Cancer Antigen (EPCA) Level and Its Association with Disease Progression in Prostate Cancer in a Chinese Population

**DOI:** 10.1371/journal.pone.0019284

**Published:** 2011-05-03

**Authors:** Zhigang Zhao, Wenjing Ma, Guohua Zeng, Defeng Qi, Lili Ou, Yeping Liang

**Affiliations:** Department of Urology, Minimally Invasive Surgery Center, The First Affiliated Hospital of Guangzhou Medical College, Guangdong Provincial Key Laboratory of Urology, Guangzhou, Guangdong Province, China; Health Canada, Canada

## Abstract

**Background:**

Early prostate cancer antigen (EPCA) has been shown a prostate cancer (PCa)-associated nuclear matrix protein, however, its serum status and prognostic power in PCa are unknown. The goals of this study are to measure serum EPCA levels in a cohort of patients with PCa prior to the treatment, and to evaluate the clinical value of serum EPCA.

**Methods:**

Pretreatment serum EPCA levels were determined with an ELISA in 77 patients with clinically localized PCa who underwent radical prostatectomy and 51 patients with locally advanced or metastatic disease who received primary androgen deprivation therapy, and were correlated with clinicopathological variables and disease progression. Serum EPCA levels were also examined in 40 healthy controls.

**Results:**

Pretreatment mean serum EPCA levels were significantly higher in PCa patients than in controls (16.84±7.60 ng/ml vs. 4.12±2.05 ng/ml, P<0.001). Patients with locally advanced and metastatic PCa had significantly higher serum EPCA level than those with clinically localized PCa (22.93±5.28 ng/ml and 29.41±8.47 ng/ml vs. 15.17±6.03 ng/ml, *P* = 0.014 and *P*<0.001, respectively). Significantly elevated EPCA level was also found in metastatic PCa compared with locally advanced disease (*P*<0.001). Increased serum EPCA levels were significantly and positively correlated with Gleason score and clinical stage, but not with PSA levels and age. On multivariate analysis, pretreatment serum EPCA level held the most significantly predictive value for the biochemical recurrence and androgen-independent progression among pretreatment variables (HR = 4.860, *P*<0.001 and HR = 5.418, *P*<0.001, respectively).

**Conclusions:**

Serum EPCA level is markedly elevated in PCa. Pretreatment serum EPCA level correlates significantly with the poor prognosis, showing prediction potential for PCa progression.

## Introduction

Prostate cancer (PCa) has emerged as the most commonly diagnosed malignancy and the second leading cause of cancer-related death among men in the great majority of Western countries [1]. Its incidence is rapidly increasing in China [2], [3]. Although the majority of patients diagnosed with PCa are currently found to have clinically localized disease [4], and local therapies with curative intent such as radical prostatectomy (RP) and radiotherapy can provide effective and durable disease control, approximately one third of those patients eventually experience biochemical recurrence [5], [6], which is often due to the early dissemination of microscopic metastatic disease that remains undetected by currently conventional pretreatment staging modalities such as radionuclide bone scan, computerized tomography (CT) scan, and magnetic resonance imaging. For locally advanced and metastatic PCa, androgen deprivation therapy (ADT) remains the most important and the primary treatment [7], which can lead to symptomatic improvement and a reduction in serum prostate specific antigen (PSA) levels in most patients. However, almost all these patients ultimately progress to hormone independence, which remains the main obstacle to improving survival and quality of life in these patients [8], [9]. In this context, it is of important interest to identify markers that are able to recognize patients prone to undergo disease progression, thereby helping to optimize therapy decisions for the individual patient. The widespread use of PSA test has led to increased detection of the disease at earlier stages and a reduction in the number of patients where metastatic disease is found at diagnosis. However, serum PSA is not independently diagnostic or prognostic in human PCa due to its significant limitations in the specificity and sensitivity [10]–[12]. Therefore, there is an urgent need for novel and improved serum prognostic biomarkers to be identified in PCa.

The nuclear matrix is responsible for maintaining nuclear shape, function, and organization of its components. The changes of its protein components have been associated with malignant transformations and translated to potential clinical applications [13]–[15]. Some studies have identified several tumor-specific nuclear matrix proteins that are associated with PCa development and correlated with poor prognostic features [16], [17]. Early prostate cancer antigen (EPCA) is a prostate cancer (PCa)-associated nuclear structural protein, which was discovered by Dhir et al in 2004 and identified as being expressed throughout the prostate of patients with PCa but not in those without the disease [18]. In the same study, the positive immunostaining for EPCA protein was also noted in the benign prostatic glands of the negative biopsies from men who were eventually diagnosed with prostate carcinoma as much as 5 years later [18]. Using an indirect enzyme linked immunosorbent assay (ELISA), Paul et al [19] firstly analyzed the plasma samples from a series of 46 individuals, including 12 PCa patients, 16 healthy donors, and 18 patients with other cancers or benign conditions. They found that mean plasma EPCA level in the patients with PCa was significantly higher than each of the other populations, and that the plasma EPCA, at a cutoff of 1.7 absorbance at 450 nm, had a sensitivity of 92% (11/12) for PCa patients, a specificity of 100% (16/16) for healthy donors, and an overall specificity of 94% (32/34) for entire donor controls. In two recent studies, we measured the EPCA levels in serum from men with isolated high-grade prostatic intraepithelial neoplasia (HGPIN) [20] and men with symptomatic benign prostatic hyperplasia (BPH) who underwent transurethral resection of the prostate (TURP) [21]. We found that elevated serum levels of EPCA were significantly correlated with increased risk of the subsequent PCa onset in HGPIN during a period of ≥5 years follow-up and the incidental PCa presence on TURP specimens analysis. All these findings suggest that up-regulation of EPCA may be involved in the presumably earlier stage of prostatic carcinogenesis and that circulating EPCA can be used as a potential diagnostic and predictive marker in human PCa. Recently, Leman et al [22] reported that the serum level of EPCA-2.22, one epitope of the EPCA-2 protein (another nuclear matrix protein different from EPCA) in patients with nonorgan-confined PCa was higher than that in those with organ-confined disease, thus ELISA assay for serum EPCA-2.22 level can accurately differentiate between organ-confined and nonorgan-confined disease. However, to the best of our knowledge, there are no published reports on serum status of EPCA and its association with clinical outcomes in PCa patient. In this study, we investigate the EPCA expression status in serum using an ELISA method in a large consecutive cohort of patients with PCa comprising clinically localized carcinomas who underwent radical prostatectomy (RP) and locally advanced and metastatic disease who received androgen-deprivation therapy (ADT) as a single treatment. We further analyzed the association of pretreatment serum EPCA level with established clinicopathological parameters of PCa, as well as the relationship between the pretreatment serum EPCA level and the onset of biochemical progression and hormone-resistant phenotype. Measurement of EPCA in blood samples could be useful for the clinical management of PCa.

## Results

### Clinical and pathological characteristics

Pretreatment data regarding initial serum PSA levels, Gleason scores, and T classification were available for all 128 studied patients. Their clinical and pathological characteristics are detailed in Table 1. In all, 77 (60.2%) patients with localised small volume cancers confined to the prostate were staged as T1-T2/N0/M0, 20 (15.6%) patients with bulky locally advanced disease staged as T3-T4/N0/M0, and 31 (24.2%) patients with evidence of metastatic spread staged as N+/M+. The mean patients age, weight, height, body mass indices, and initial PSA level were 63.3±5.7 years (median: 65 years, range: 46–78 years), 74.2±5.1 kg (median: 76 kg, range: 58–84 kg), 170.2±5.0 cm (median: 172 cm, range: 155–183 cm), 23.44±2.82 kg/m^2^ (median: 21.67 kg/m^2^, range: 17.62–28.21 kg/m^2^), and 12.64±6.17 ng/ml (median: 10.56 ng/ml, range: 2.20–130.84 ng/ml), respectively. The mean age of healthy control group was 64.1±6.0 years (median: 66 years, range: 45–80 years).

**Table 1:** Clinical and Pathologic Features of 128 Patients with PCa.

Characteristics	N (%)
**Initial serum PSA levels**	
0–2.5	5 (3.9)
2.6–4.0	16 (12.5)
4.1–9.9	40 (31.3)
≥10.0	67 (52.3)
**Gleason score**	
2–4	16 (12.5)
5–6	68 (53.1)
7	24 (18.8)
8–10	20 (15.6)
**Clinical TNM stage**	
T1N0M0	20 (15.6)
T2N0M0	57 (44.5)
T3N0M0	7 (5.5)
T4N0M0	13 (10.2)
T(any)N+M0	10 (7.8)
T(any)N(any)M+	21 (16.4)

### Follow-up results

No patient was lost to follow-up. All patients had regular PSA measurements from the time of surgery or the initiation of ADT through the follow-up period. The mean and median length of follow-up was 39.4 and 35.8 months (range: 2–72 months), respectively. For 77 prostatectomy patients, 19 (24.7%) cases developed the biochemical progression during the follow-up. The mean and median time from RP to biochemical recurrence were 27.4 and 24.5 months (range: 2–49.9 months), respectively. Among the 51 patients with locally advanced and metastatic PCa who were treated by ADT as the primary treatment, 39 (76.5%) cases were identified as having AIP after the initiation of ADT. The mean and median time from the ADT initiation to AIP in this cohort were 25.8 and 21.3 months (range: 6–52 months), respectively.

### Baseline serum levels of EPCA in healthy controls and PCa patients

EPCA was detectable in the sera from all healthy control subjects and PCa patients. The measurement results of serum EPCA in normal healthy controls and PCa patients are shown in Table 2. Obviously, the mean serum EPCA levels were significantly different between the healthy subjects and cancer patients (P<0.001). When the results from the three groups of patients with clinically localized PCa (T1-T2), locally advanced PCa (T3-T4), and metastatic disease were individually compared with the controls, serum EPCA levels were significantly higher in any of the three groups (all Ps<0.001). Moreover, patients with metastatic PCa had significantly higher serum EPCA levels than those in the both groups of patients with clinically localized PCa and locally advanced disease (P<0.001 and P<0.001, respectively). Significantly elevated levels of EPCA were also found in patients with locally advanced PCa as compared with the clinically localized PCa (P = 0.004). In addition, serum EPCA levels between PCa patients with lymph node metastases and distant organ metastases (bone and liver) was not significantly different (P = 0.28). Likewise, two groups of ADT treated patients who received LHRH agonists plus antiandrogen agents and who received bilateral orchidectomy plus antiandrogen agents had similar serum EPCA levels (P = 0.31).

**Table 2:** Baseline serum levels of EPCA in healthy controls and PCa patients and association of EPCA levels with clinicopathological variables in 128 prostatic carcinomas.

Characteristics	Mean±SD (ng/ml)	Median (ng/ml)	Range (ng/ml)
**Group**			
Healthy controls (n = 40)	4.12±2.05	3.35	0.68- 8.77
PCa patients (n = 128)	16.84±7.60	15.20	5.20–45.82
p Value§	<0.001*		
**Age (year)** ∥			
≤65	17.82±7.50	14.95	5.20–35.30
>65	19.03±8.28	16.19	10.35–45.82
p Value§	0.102*		
**Clinical stage**			
T1-T2	15.17±6.03	13.85	5.20–20.25
T3-T4	22.93±5.28	19.80	13.60–40.00
p Value§	<0.001*		
**Gleason score**			
2–6	13.36±4.02	12.00	5.20–21.00
7	21.61±8.11	18.53	12.90–35.30
8–10	31.08±9.24	28.25	20.25–45.82
p Value§	<0.001†		
**Initial serum PSA level (ng/ml)**			
≤10	19.50±8.42	17.72	10.35–45.82
>10	18.74±7.60	16.90	5.20–35.30
p Value§	0.274*		
**Metastatic status** ‡			
Yes	29.41±8.47	28.00	21.00–45.82
No	14.83±6.25	12.90	5.20–23.43
p Value§	<0.001*		

∥ Categorized by the median value.

*Mann-Whitney U test.

† Kruskal-Wallis test.

‡ Including lymph node metastases and distant metastases to bone and liver.

§ p<0.05, statistically significant.

### Association of pretreatment serum EPCA levels with clinicopathologic characteristics of PCa Patients

Table 2 also shows the correlation between EPCA levels and clinicopathologic features in patients with PCa. Pretreatment serum EPCA levels were significantly elevated in PCa patients with higher Gleason score (P<0.001), clinical advanced stage (P<0.001), and metastatic status (P<0.001). From the Kruskal–Wallis test, the pretreatment serum EPCA level was significantly and positively correlated with tumour Gleason score. The Mann-Whitney test also showed a significant and positive correlation between the serum EPCA expression level and clinical stage. Tumours with Gleason score 7–10 or stage T3-T4 tended to have a significantly higher serum EPCA expression level than those with Gleason score ≤6 or T1-T2 stage. Furthermore, we also found that pretreatment serum EPCA level was significantly associated with metastatic disease. PCa patients with metastases tended to have a significantly higher EPCA expression level in their sera than those with localized carcinomas. To evaluate the association between pretreatment serum EPCA levels and the disease progression, we assessed the changes of serum EPCA levels at the time points of presentation prior to treatment between patients with disease progression and those without. Analysis of the measurements revealed that all patients with progressive disease had significantly higher levels of serum EPCA (mean: 28.55±5.76 ng/ml, range: 19.20–45.82 ng/ml) than those without progression (mean 13.82±4.50 ng/ml, range: 5.20–22.45 ng/ml, P<0.001). However, the mean serum EPCA level in patients with biochemical progression after RP (27.85±6.14 ng/ml, range: 19.20–43.60 ng/ml) was not significantly different from that in those with AIP after the initiation of ADT (30.36±7.52 ng/ml, range: 21.00–45.82 ng/ml, P = 0.280).

The initial serum PSA levels in this cohort were compared with their pretreatment serum EPCA levels. However, no statistically significant correlation between serum EPCA and PSA levels was identified (P = 0.274). Although the elder men (>65 years) appeared to have an increased serum EPCA level, the serum EPCA level did not statistically correlate with patient age (P = 0.102). The univariate analysis further demonstrated that pretreatment serum EPCA level was not correlated with initial serum PSA levels (correlation coefficient = 0.148, P = 0.173) and patients age (correlation coefficient = 0.082, P = 0.316).

### Association of pretreatment serum EPCA levels with biochemical progression after RP

**Figure 1:**
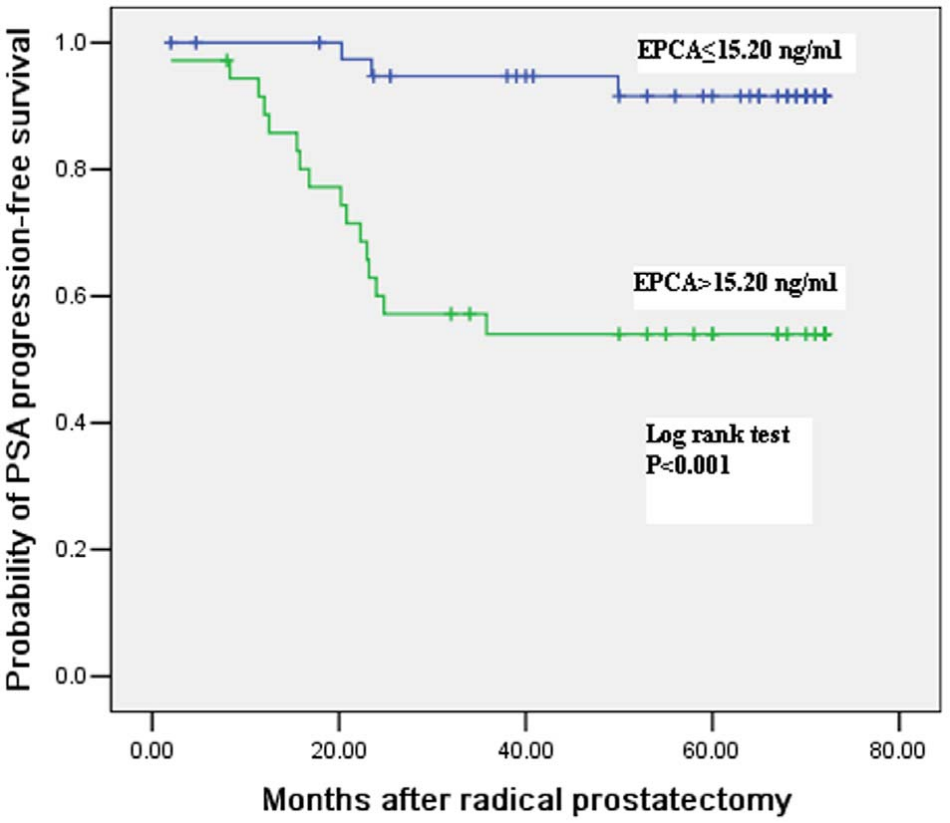
Kaplan-Meier estimates of PSA progression-free survival probability for the 77 patients with clinically localized PCa treated with RP, who were grouped by the baseline serum EPCA level above or below the median value of 15.20 ng/ml.

To further assess the relationship between pretreatment serum EPCA levels and the onset of biochemical recurrence after RP, we performed a Kaplan-Meier progression-free actuarial probability curve in the 77 patients with clinically localized PCa who were treated with RP. As shown in Figure 1, the Kaplan-Meier analysis revealed that patients with elevated pretreatment serum EPCA levels (greater than the median value of 15.20 ng/ml) had an increased probability of PSA progression (log-rank test, P<0.001). At a mean follow-up of 39.4 months, the biochemical progression-free survival probability of patients with clinically localized PCa was 52.8% in 36 cases with pretreatment serum EPCA level >15.20 ng/ml in the serum in comparison with the rate of 95.1% in the remaining 41 cases with pretreatment serum EPCA level ≤15.20 ng/ml (*P*<0.001). Moreover, the mean time from RP to biochemical recurrence in patients with pretreatment serum EPCA levels >15.20 ng/ml (20.4 months, range: 2–35.8 months) was significantly shorter than that in those with pretreatment serum EPCA levels ≤15.20 ng/ml (36.7 months, range: 23.5–49.9 months, *P*<0.001). On the univariate analysis, serum EPCA level was significantly associated with the risk of PSA progression following RP (Table 3, *P*<0.001), along with Gleason score (Table 3, *P* = 0.016). On a preoperative multivariate model, the association between serum EPCA level and biochemical progression remained statistically significant (Table 3, *P*<0.001), and the HR was 4.860 (Table 3, 95%CI: 2.070–6.035) for pretreatment serum EPCA. However, in the same multivariate analysis, the Gleason sum had not independent prognostic value of the biochemical progression (Table 3). These data demonstrate that pretreatment serum EPCA is a significant predictor for the development of biochemical progression postoperatively in patients with clinically localized PCa.

**Table 3:** Univariate and multivariate Cox regression analyses of pre-operative variables for the prediction of biochemical progression after RP for 77 patients with clinically localized PCa.

	Univariate			Multivariate		
Variables	Hazard Ratio	95%CI	p Value§	Hazard Ratio	95%CI	p Value§
Preoperative EPCA levels	5.232	3.172–7.145	<0.001	4.860	2.070–6.035	<0.001
Initial PSA levels*	2.633	1.214–4.115	0.083	1.825	0.738–2.924	0.140
Clinical stage†	1.527	0.653–2.431	0.172	0.818	0.374–1.623	0.202
Gleason score‡	3.044	1.242–4.329	0.016	2.102	1.053–3.720	0.114

*Initial PSA levels were categorized as ≥10 ng/ml versus <10 ng/ml.

† Clinical stage was categorized as T1 versus T2.

‡ Gleason score was categorized as grade 2 to 6 versus grade 7 to 10.

§ p<0.05, statistically significant.

### Association of pretreatment serum EPCA levels with androgen-independent progression

**Figure 2:**
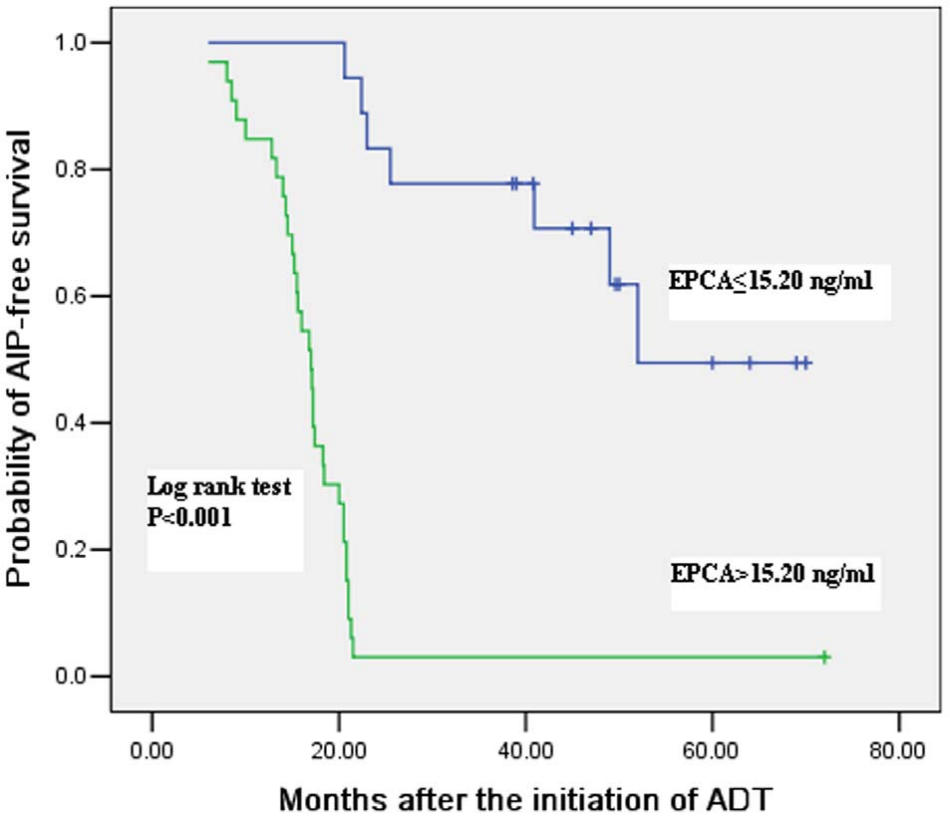
Kaplan-Meier estimates of AIP-free survival probability for the 51 patients with locally advanced and metastatic PCa treated with ADT, who were grouped by the baseline serum EPCA level above or below the median value of 15.20 ng/ml.

To further assess the relationship between pretreatment serum EPCA levels and the appearance of AIP, we performed a Kaplan-Meier AIP-free actuarial probability curve in the 51 patients with locally advanced or metastatic PCa who were treated with ADT as the sole treatment. The Kaplan-Meier analysis revealed that patients with pretreatment serum EPCA level >15.20 ng/ml had a significantly increased probability of the development of AIP (Figure 2, log-rank test, P<0.001). Following a mean follow-up of 39.4 months (range: 2–72 months) after the initiation of ADT, the AIP-free survival probability was 9.3% for 33 men with pretreatment serum EPCA level >15.20 ng/ml compared to the rate of 61.1% for the rest 18 men with serum EPCA level ≤15.20 ng/ml (*P*<0.001). The mean time for achieving a status of AIP was 12.4 months (range: 6–21.5 months) in patients with serum EPCA level >15.20 ng/ml and 35.2 months (range: 20.6–52 months) in patients with serum EPCA level ≤15.20 ng/ml, respectively. The Kaplan–Meier curve demonstrated that patients with pretreatment serum EPCA level ≤15.20 ng/ml had a longer time for the presence of AIP than those with serum EPCA level >15.20 ng/ml (P<0.001). Furthermore, an univariate Cox proportional hazard model analysis showed that those patients with elevated serum EPCA level prior to the ADT had a significantly increased risk of the onset of AIP compared with those with pretreatment serum EPCA≤15.20 ng/ml (Table 4, *P*<0.001). Also, the initial PSA level and the clinical stage were significantly correlated with the risk of AIP (Table 4). On a multivariate model, the pretreatment serum EPCA level remained a statistically significant association with the presence of AIP after androgen suppression, with a HR of 5.418 (Table 4, 95%CI: 3.637–7.251, P<0.001), whereas the other variables like the clinical stage and the initial PSA level did not (Table 4). These data demonstrate that pretreatment serum EPCA is a significant predictive variable for the development of AIP in patients with locally advanced and metastatic disease.

**Table 4:** Univariate and multivariate Cox regression analyses of pre-treatment variables for the prediction of AIP after ADT for 51 patients with locally advanced or metastastic PCa.

	Univariate			Multivariate		
Variables	Hazard Ratio	95%CI	p Value§	Hazard Ratio	95%CI	p Value§
Preoperative EPCA levels	6.825	3.702–8.436	<0.001	5.418	3.637–7.251	<0.001
Initial PSA levels*	2.702	1.106–4.035	0.025	1.213	0.482–2.428	0.140
Clinical stage†	3.533	2.146–5.126	0.002	1.626	0.759–3.035	0.108
Gleason score‡	2.237	1.002–3.724	0.114	1.051	0.442–2.132	0.262

*Initial PSA levels were categorized as ≥10 ng/ml versus <10 ng/ml.

† Clinical stage was categorized as T3-T4 versus N+/M+.

‡ Gleason score was categorized as grade 2 to 6 versus grade 7 to 10.

§ p<0.05, statistically significant.

## Discussion

To date, no validated serum biomarker has been identified with prognostic power in human PCa. In this study, using an ELISA test, we demonstrated a significantly elevated serum EPCA level in patients with PCa in comparison with the normal healthy men without cancer. We found that patients with metastatic PCa had a significantly higher EPCA level in serum than those with clinically localized and locally advanced PCa, respectively. Also, in this prospective cohort of PCa patients, the serum EPCA level of locally advanced PCa patients was significantly higher than that of clinically localized PCa patients. These data suggest an association of serum EPCA with the malignant phenotype of prostatic carcinoma.

Thus, we studied the association between serum EPCA levels and clinicopathological features of PCa patients. In the present 128 patients, pretreatment serum EPCA levels were significantly and positively correlated with the clinical risk factors of poor prognosis such as Gleason score and stage. We found that patients with high Gleason score (≥7) or advanced clinical stage (T3-T4 or N+/M+) tended to have higher serum EPCA levels prior to the treatment. These findings are not in accordance with previous immunohistochemical studies, where the significant and positive correlation between EPCA expression levels in PCa tissue and tumor stage was not found [18], [28]. This discrepancy may be related to the different sensitivity of the assays and the difference of the protein levels expressed in blood and tissue. In a previous study on circulating EPCA levels in PCa patients performed by Paul and coworkers [19], the plasma EPCA levels was not significantly correlated with Gleason grade because the Gleason Score was either 6 or 7 in all but one patient, whereas the association between the plasma EPCA level and tumor stage was not examined. In the same study, they also reported that no statistically significant correlation was identified between the plasma EPCA level and PSA level because most of the PSA levels fell within the clinically indistinguishable range of 4–10 ng/ml. Concordantly with this result, we found in the present study that the serum EPCA levels were not correlated with initial serum PSA levels. Our findings suggest that serum levels of EPCA expression are positively associated with tumor burden independently of serum PSA. The studies described here were not designed to examine the serum EPCA levels after RP or ADT but from the recent reports that serum level of EPCA-2.22 after RP was significantly lower than that before surgery (*P*<0.001) and considered to be normal postoperatively [22], it would seem that the levels of serum EPCA fall following the ablation of the prostatic cancer. The previous reports have shown that patients age probably does not influence EPCA levels in the plasma in a relatively small-sized population [19]. To address whether the observed elevation of serum EPCA level in patients with PCa was age dependent, we compared their median age (and range) with that of the healthy controls. We found that the median age (and range) was not significantly different from each other. Moreover, PCa patients of age >the median value of 65 years had the similar EPCA level in serum to those of age ≤65 years (P = 0.102). Taken together, we can exclude the possibility of age-dependent expression of serum EPCA in patients with PCa. Additionally, the serum EPCA levels were not significantly different between patients with lymph node metastases and distant organ metastases, indicating that baseline serum EPCA levels were independent of the metastatic sites.

Furthermore, we investigate the correlation of pretreatment serum concentrations of EPCA with PCa progression to biochemical relapse or androgen-independence. The results suggested that disease progression may occur earlier in those PCa patients with an elevated pretreatment serum EPCA level, and that the elevated EPCA level in serum may be an early event during the disease progression of human PCa. Initial serum PSA level, tumor Gleason score, and clinical stage are pretreatment factors related to clinical risk of human PCa. Some authors reported that these variable did not predict the interval to disease progression [26], [29]. Concordantly with these authors, we found that initial PSA level, Gleason grade, and clinical stage were not associated with cancer progression in the present study. Although the univariate analysis showed that the tumor Gleason score had significance in predicting the biochemical progression after RP in patients with clinically localized PCa, and that the initial PSA level and tumor clinical stage were significantly predictive of the time to AIP in patients with locally advanced or metastatic PCa under androgen suppression as a single therapy, all these variables lost their statistical significance on the multivariate analyses. In this cohort of patients, the multivariate Cox proportional hazards models demonstrated that the elevated pretreatment serum EPCA held the most significantly predictive value for the disease progression among these pretreatment factors.

The highly variable nature of PCa progression constitutes a major problem in clinical management of the individual patient. The identification of more validated markers that advance the ability to predict the clinical progression and prognostic outcome of the disease would help to optimize therapy decisions. In addition, with the continued widespread use of PSA-based screening, current patients who undergo RP have lower levels of PSA, which further supports the need for novel serum markers that better predict disease outcome in these patients. Our results showed that the elevated serum EPCA level can be detected significantly sooner than the presence of biochemical recurrence after radical surgery in clinically localized PCa and the oneset of AIP after ADT in locally advanced or metastatic PCa. Based upon those data, there are at least two potential clinical benefits to the identification of pretreatment serum EPCA levels. The first is prognostic: higher pretreatment serum EPCA level may add useful prognostic information above and beyond traditional clinical risk factors for the disease progression. Therefore, measurement of serum EPCA levels with ELISA before RP has the potential to identify patients at high risk of postoperatively biochemical recurrence, thereby provides timely information to guide postoperative neoadjuvant or adjuvant intervention. Also, higher serum EPCA level in PCa patients beginning ADT may provide important and unique information. For example, a study of chemotherapy in castration-sensitive prostate cancer might limit eligibility to patients with elevated serum EPCA levels present to enrich for a particularly poor prognostic cohort. The second is therapeutic: based upon the results presented here, we suggest that the analysis of EPCA in serum might help to identify patients who benefit from early multimodal therapy and might facilitate a new therapeutic strategy that may compensate for current limitations of diagnosis based on serum PSA alone. In patients with presumed organ-confined PCa, the measurement of serum EPCA before RP may alter the surgical approach. Nerve sparing RP has been recently used to preserve erectile function. With this approach the distance between the prostatic capsule and surgical margin is significantly decreased. Studies have addressed the potential cure rate when wider surgical excision of the neurovascular bundle is performed in patients with extraprostatic tumor [30], [31]. Therefore, preoperative measurement of serum EPCA by ELISA may help identify cases in which nerve sparing RP should not be considered due to the presence of extraprostatic tumor extension.

Our study was limited by the small-sized sample. The follow-up time was relatively short (median: 35.8 months) in the present study, nonetheless, our results have significant clinical implications for human PCa because the risk of cancer specific mortality is especially greater in patients with PSA recurrence within the first 3 years after RP [32]. In addition, there were relatively many patients with advanced PCa in this study (extraprostatic in 40% of the patients). Because there is no PSA-based screening programme for PCa in China, more patients are diagnosed with locally advanced and/or metastatic disease than in countries with PSA screening [2], [3]. Thus, there could be differences if our data are transferred to other populations, especially in a screened population. Despite the limitations of this hospital based cohort study, our results demonstrate the potential of serum EPCA as a prognostic predictor in PCa progression by phenotype manifestation in survival analysis. If the findings presented here are corroborated by larger population cohort studies and in more races, EPCA may become an important biomarker and an indicator for distinct therapy options.

In summary, this prospective study investigates the pretreatment EPCA level in serum and its association with clinical outcome in a cohort of patients with PCa. The pretreatment serum EPCA levels are markedly elevated in PCa patients, and significantly correlated with high Gleason grade and clinical advanced stage independently of the serum PSA levels and patients age. Furthermore, our results add to the literature by documenting the significance of baseline serum EPCA level as a distinct predictor of disease progression comprising biochemical recurrence and AIP in human PCa. Although extensive clinical trials are required to confirm these findings, this initial study shows that measurement of pretreatment serum EPCA may be of clinical importance and may help to identify cancer patients at increased risk of tumor progression, thereby optimizing therapeutic decisions for the individual patient.

## Materials and Methods

### Patient population and treatment

From July 2004 to July 2010, we prospectively evaluated 128 consecutive patients with prostatic adenocarcinoma who were diagnosed and treated at Minimally Invasive Surgery Center (MISC), Guangzhou Medical College. All the patients were staged by digital rectal examinations (DRE) and transrectal ultrasound (TRUS) for local disease and by bone scanning and CT of the abdomen and pelvis for metastatic disease according to the 2002 American Joint Committee on Cancer (AJCC) TNM classification criteria [23]. All patients had histopathological confirmation of the diagnosis of PCa based on examination of prostatic tissues obtained by TRUS-guided prostate biopsy or by TURP. The histologic grade of the tumor was determined using the Gleason's score system [24]. Of these 128 patients, 77 men (60.2%) had clinically localized PCa and were treated by retropubic radical prostatectomy (RP). The remaining 51 patients (39.8%) were diagnosed as having locally advanced (n = 20) and metastatic PCa (n = 31). The metastatic disease included lymph node metastases in 10 patients, distant metastases to bone in 19 and to liver in 2 patients. All the 51 patients received androgen-deprivation therapy (ADT) as the sole treatment. The type of ADT was chemical [i.e. luteinizing hormone–releasing hormone (LHRH) agonists plus antiandrogen agents] in 27 patients and surgical (i.e. bilateral orchidectomy plus antiandrogen agents) in the remaining 24 patients. All patients had no evidence of active infection or inflammatory disease. No patients were treated before collecting blood samples.

This study also included 40 normal healthy men without cancer, who were presenting for prostatic cancer screening at our MISC. They had no prior history of cancer and chronic disease, normal DRE findings, and a PSA level <2.0 ng/ml, which is in the PSA range with an estimated PCa detection probability of less than 1% in the first 4 years after screening [25]. Prior to commencing this study, the approval from the Ethics Review Board of Guangzhou Medical College was granted and the written informed consent was obtained from each patient.

### Serum samples and EPCA measurement

Blood samples were prospectively collected from 128 PCa patients by drawing on the morning of the scheduled day of RP or on the morning of the starting day of ADT after a pretreatment overnight fast. For patients who underwent TRUS-guided needle biopsy of the prostate, the blood samples were drawn at least 4 weeks after the biopsy. The blood samples from 40 normal healthy men were also drawn on the morning of the scheduled day of visiting after a overnight fast. The samples were collected into evacuated tubes, and serum was separated within 1 h of blood collection after centrifuging at 3,000 rpm for 10 min at room temperature. The serum was subsequently decanted using sterile transfer pipettes and stored immediately at −80°C until analysis.

We have established the optimal conditions for the indirect ELISA for the measurement of serum EPCA protein in a pilot study [21]. The specificity and sensitivity for serum EPCA in discriminating PCa were 98.0% and 100%, respectively [21]. In the present study, a commercially available anti-EPCA polyclonal antibody (Onconome Inc., Seattle, Washington, USA) was used, and the procedure of ELISA for EPCA measurement was performed as described previously [21]. Briefly, serum samples from each patient were plated in triplicate in 96-well Nunc Immunoplate MaxiSorb plates (Nunc, Wiesbaden, Germany). When the reaction was stopped, the plates were read at 450 nm on the LabSystems Multiskan Multisoft microplate reader (LabSystems, Helsinki, Finland). The concentration that corresponds to the absorbance level in each sample was calculated from the standard curve equation. The mean of the three measurements for each sample were used for data analyses. The differences among the measurements were minimal, with the intra-assay precision coefficients of variation of <10%. In this study, all assays were performed by an experienced laboratory expert at our institute (L.O.), who was blinded with respect to the case status of the samples. Elevated serum level of the EPCA was defined as being greater than the median value of 15.20 ng/ml in this studied cohort of patients.

### Follow-up and definitions of progression

All patients were continuously seen by the urologists in our Department from the date of RP or ADT. Each of the 77 patients who underwent RP was scheduled to undergo a DRE and assay of serum PSA level starting 4 weeks postoperatively, every 3 months for the first postoperative year, semiannually from the second through the fifth year, and annually thereafter. In the 51 patients with locally advanced or metastatic PCa who received ADT, serum PSA was determined every three months until androgen-independent progression (AIP) occurred. The primary outcome variables were biochemical progression after RP for patients with clinically localized PCa and time to AIP during treatment with ADT for those with locally advanced or metastatic disease. Biochemical progression was defined as a sustained elevation of serum PSA level >0.2 ng/ml on two or more occasions, and the date of progression was assigned as the date of the first PSA increase value >0.2 ng/ml. The AIP was defined as two consecutive rises of serum PSA (at least 1 week apart) greater than the nadir value while receiving ADT. The nadir PSA was defined as the minimal serum PSA observed during the follow-up [26]. The time to the AIP during ADT was defined as the duration of time from the initiation of ADT to the date of AIP or was censored among patients who did not progress at the date last known progression free or the date of death who died without progression [7], [27]. In this study, disease progression was defined as either the appearance of biochemical progression in clinically localized PCa or the onset of AIP in locally advanced or metastatic PCa. The overall survival time was calculated for each patient to the nearest month, taken from the time of presentation to the time of death/last follow-up. Pretreatment and posttreatment serum PSA concentrations for each patient were measured always by using a Hybritech Access® PSA assay (Beckman Coulter, Inc., Fullerton, CA).

### Statistical analysis

Patient clinical characteristics were summarized as number and percentage of patients or median and range of values. The association between the serum EPCA levels and different clinicopathological features was assessed by use of the Mann-Whitney test and the Kruskal-Wallis test, as appropriate. The Kaplan-Meier method was used to calculate survival functions, and differences were assessed by log-rank analysis. Univariate and multivariate survival analyses were performed with the Cox proportional hazards regression model. The hazard ratio (HR) with 95% confidence interval (CI) was calculated to determine the power of parameters for predicting the disease progression. Pretreatment parameters were assessed in terms of pretreatment serum EPCA level, initial PSA level, Gleason score, and clinical stage. Pretreatment EPCA level was used as a continuous variable, which was dichotomized at the median value within the cohort. Clinical stage was entered as a categorical variable and evaluated as T1–2 versus T3–4. Gleason score were entered as categorical variables and evaluated as grade 2 to 6 versus grade 7 to 10. All reported P values represent two-sided tests of statistical significance. In this study, a P<0.05 was considered as statistically significant. All statistical analyses were performed with the Statistical Package for the Social Sciences (SPSS) statistical software package version 13.0 for Windows (SPSS, Inc, Chicago, IL, USA).
